# Supplementary material for: Integrated chemical and genetic screens unveil FSP1 mechanisms of ferroptosis regulation
Source: Nat Struct Mol Biol. 2023 Nov 13;30(11):1806–15. doi: 10.1038/s41594-023-01136-y (PMC10643123; doi:10.1038/s41594-023-01136-y)

nature structural & molecular biology

Article

https://doi.org/10.1038/s41594-023-01136-y

Integrated chemical and genetic screens unveil FSP1 mechanisms of ferroptosis regulation

In the format provided by the
authors and unedited

Supplementary Fig. 1

An example of the gating strategy for lipid peroxidation assay using C11-BODIPY

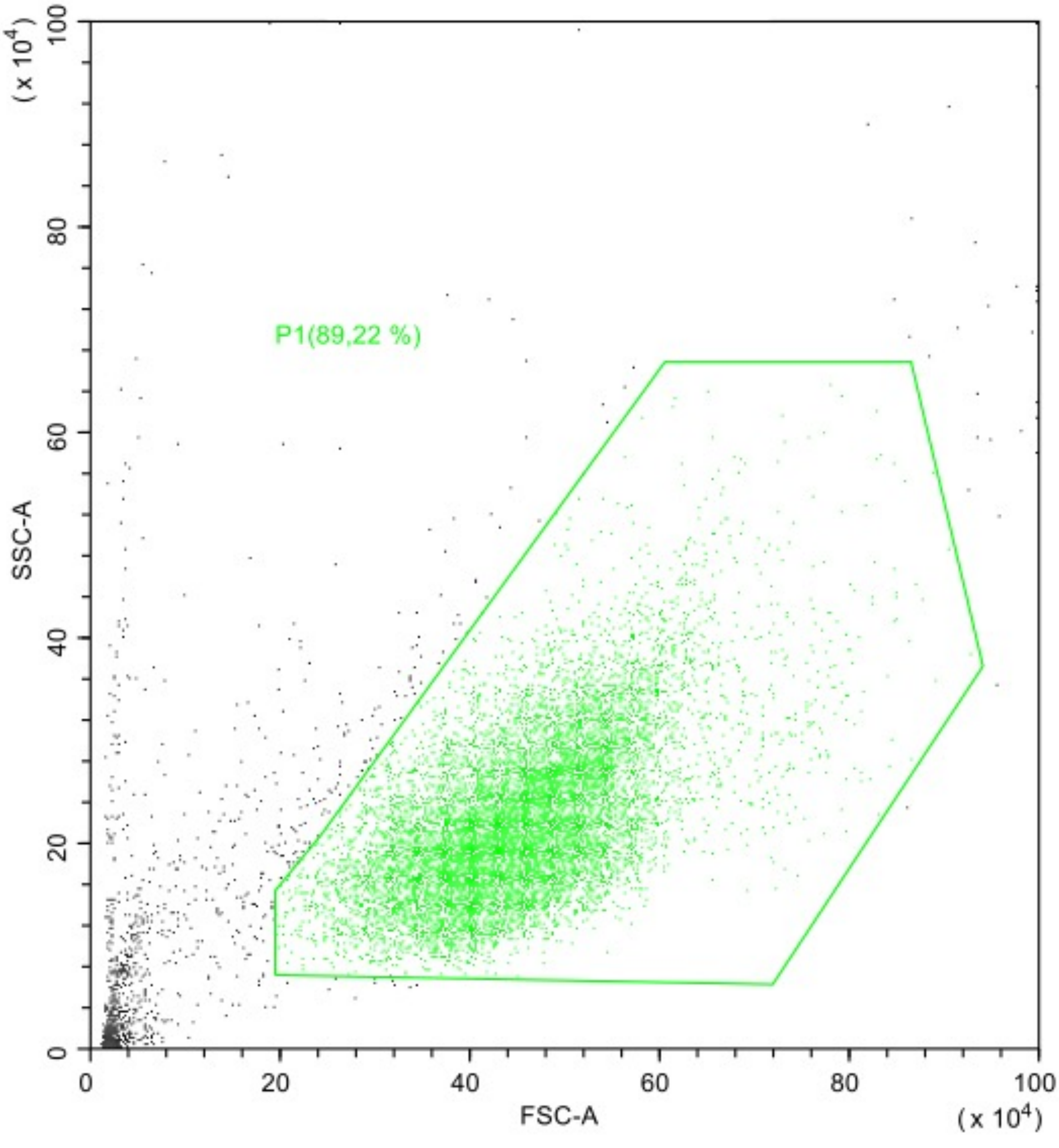

Supplementary Fig.1

An example of the gating strategy for lipid peroxidation assay using C11-BODIPY
Green area was considered as viable cells and analyzed.

Supplementary Fig. 2

Extended data Fig. 2c

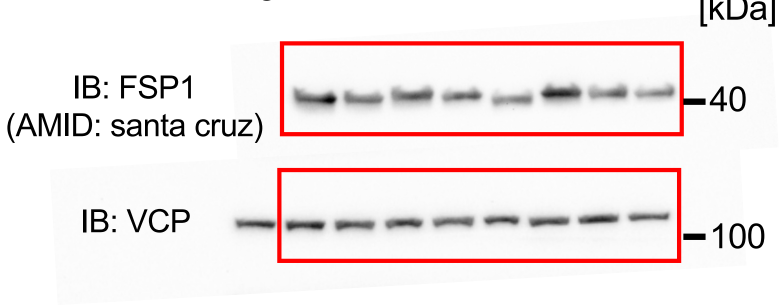

Extended data Fig. 3a

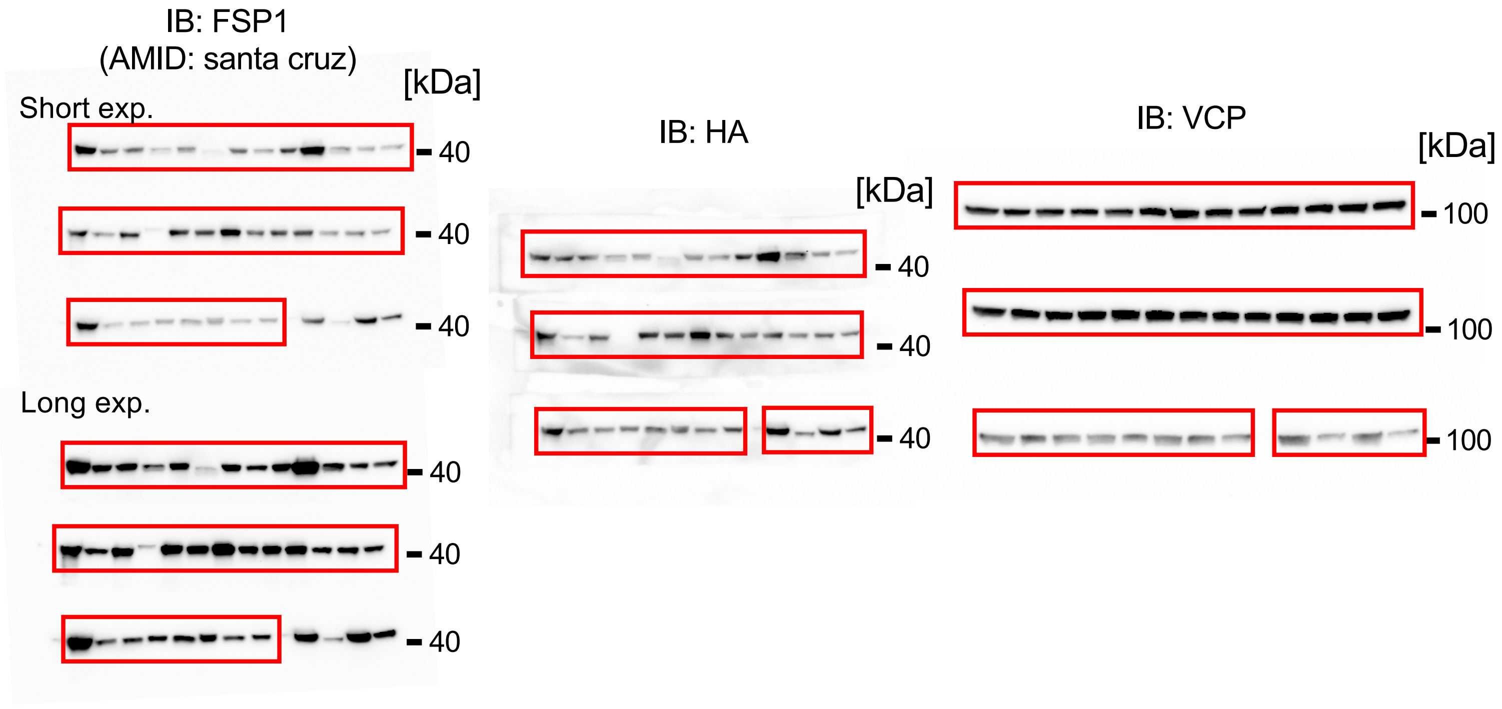

Extended data Fig. 3e

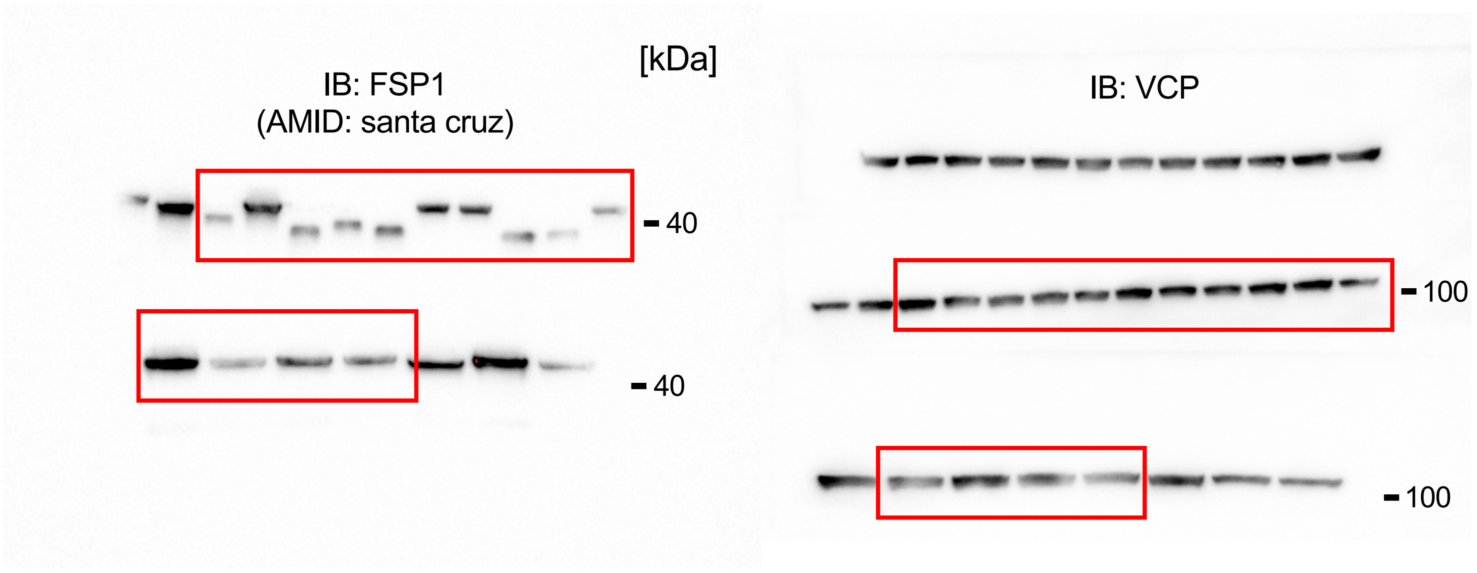

Supplementary Fig.2

Raw images of immunoblotting with a molecular marker were shown.
Red area were cropped for visualization.

Supplementary Fig. 2

Extended data Fig. 4a

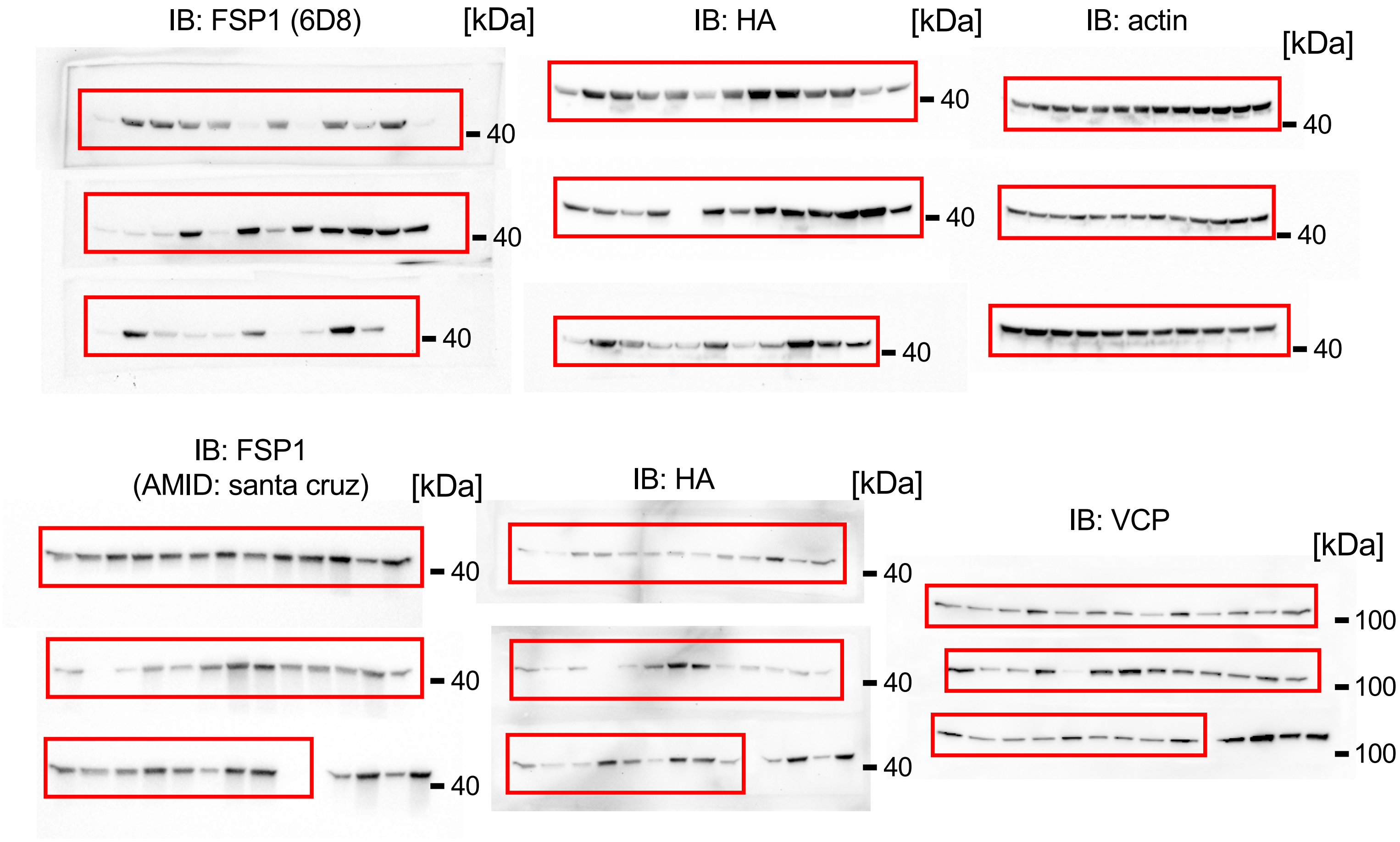

Extended data Fig. 4b& 7d

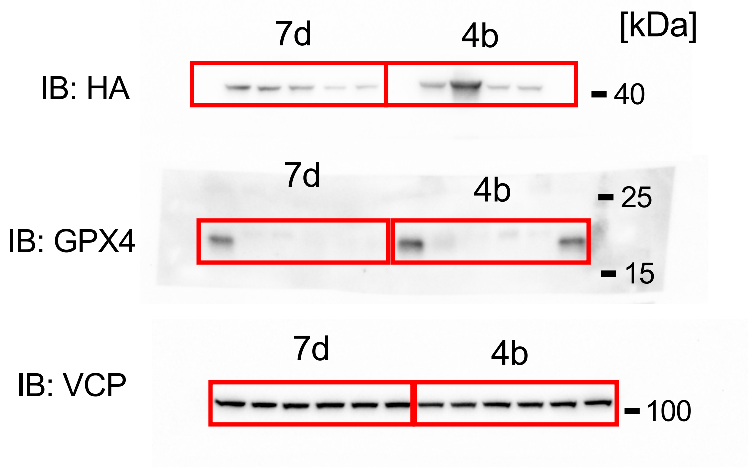

Extended data Fig. 4g

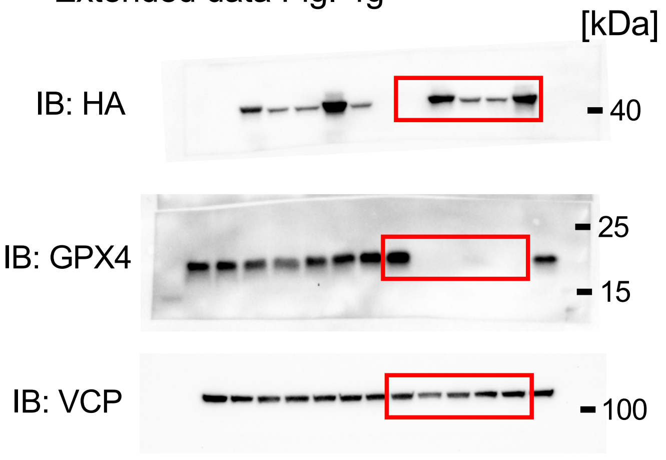

Extended data Fig. 4c

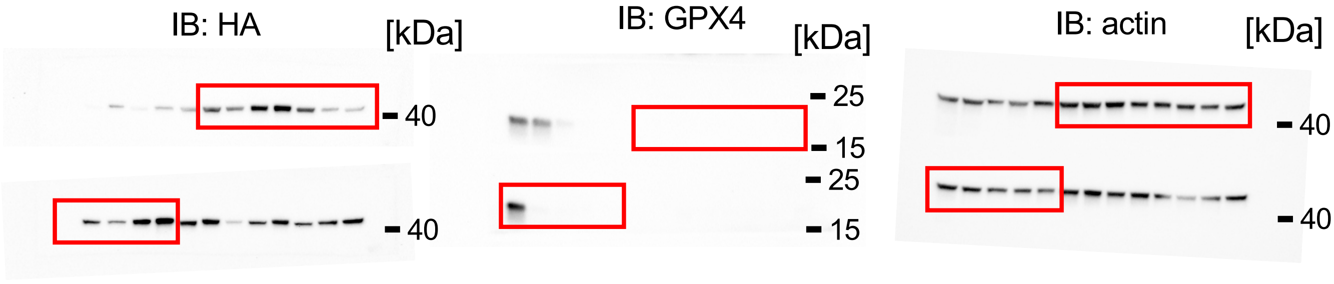

Supplementary Fig.2 (continued)

Extended data Fig. 6c

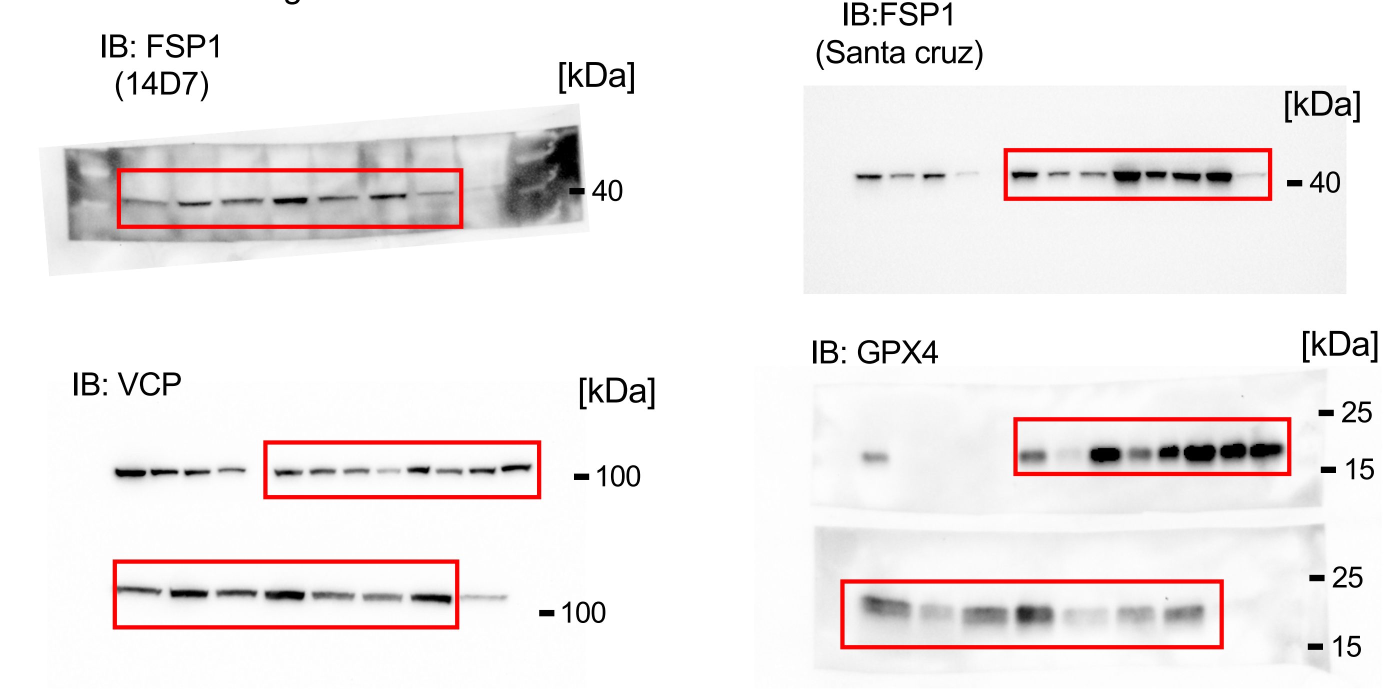

Extended data Fig. 6e (left)

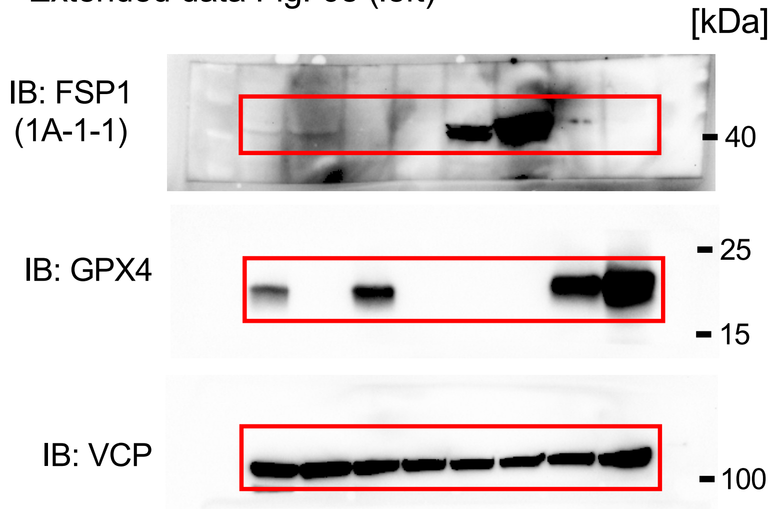

Extended data Fig. 6e (right)

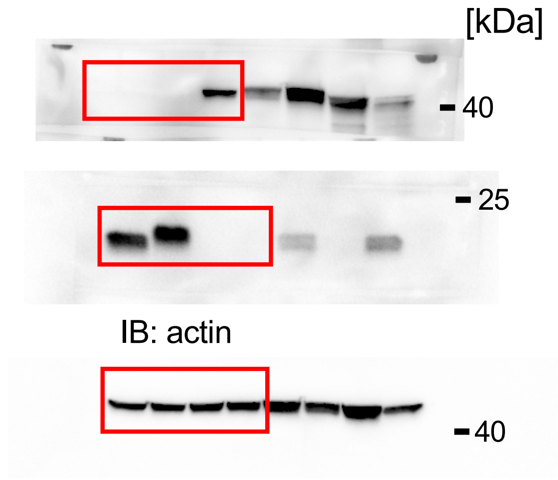

Extended data Fig. 6e (middle)

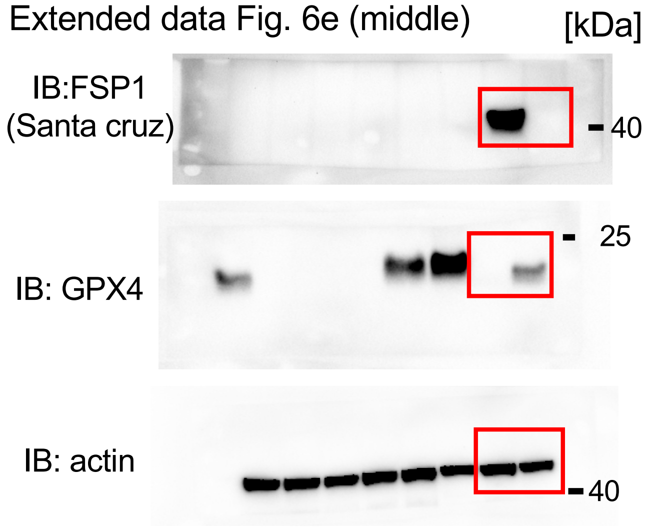

Extended data Fig. 6h

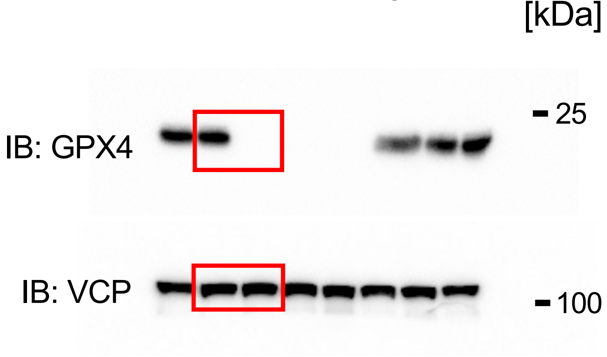

Supplement: Supplementary file 1 — Supplementary Figures 1 and 2 [file 41594_2023_1136_MOESM1_ESM.pdf]
