# Supplementary material for: Integrated chemical and genetic screens unveil FSP1 mechanisms of ferroptosis regulation
Source: Nat Struct Mol Biol. 2023 Nov 13;30(11):1806–15. doi: 10.1038/s41594-023-01136-y (PMC10643123; doi:10.1038/s41594-023-01136-y)
Supplement: Supplementary file 2 — Reporting Summary [file 41594_2023_1136_MOESM2_ESM.pdf]

nature portfolio

Corresponding author(s): Marcus Conrad

Last updated by author(s): Aug 31, 2023

Reporting Summary

Nature Portfolio wishes to improve the reproducibility of the work that we publish. This form provides structure for consistency and transparency in reporting. For further information on Nature Portfolio policies, see our Editorial Policies and the Editorial Policy Checklist.

Statistics

For all statistical analyses, confirm that the following items are present in the figure legend, table legend, main text, or Methods section.

n/a	Confirmed	
☐	☒	The exact sample size (*n*) for each experimental group/condition, given as a discrete number and unit of measurement
☐	☒	A statement on whether measurements were taken from distinct samples or whether the same sample was measured repeatedly
☐	☒	The statistical test(s) used AND whether they are one- or two-sided<br>*Only common tests should be described solely by name; describe more complex techniques in the Methods section.*
☒	☐	A description of all covariates tested
☐	☒	A description of any assumptions or corrections, such as tests of normality and adjustment for multiple comparisons
☐	☒	A full description of the statistical parameters including central tendency (e.g. means) or other basic estimates (e.g. regression coefficient) AND variation (e.g. standard deviation) or associated estimates of uncertainty (e.g. confidence intervals)
☐	☒	For null hypothesis testing, the test statistic (e.g. *F*, *t*, *r*) with confidence intervals, effect sizes, degrees of freedom and *P* value noted<br>*Give P values as exact values whenever suitable.*
☒	☐	For Bayesian analysis, information on the choice of priors and Markov chain Monte Carlo settings
☒	☐	For hierarchical and complex designs, identification of the appropriate level for tests and full reporting of outcomes
☒	☐	Estimates of effect sizes (e.g. Cohen's *d*, Pearson's *r*), indicating how they were calculated

Our web collection on statistics for biologists contains articles on many of the points above.

Software and code

Policy information about availability of computer code

Data collection	CytExpert v2.4 (Beckman Coulter), Eve v1.8.2 (Nanolive), Image Lab v6.0 (Biorad), SoftMax Pro v7 (Molecular Devices).
Data analysis	Flow Jo v10 software (Treestar, Inc), GraphPad Prism v9 (GraphPad Software), Image J/Fiji v1.53 (NIH), Burrows-Wheeler Alignment Tool (v0.7.17-r1188), MuSyC (https://musyc.lolab.xyz), FAstQC (v0.11.7), Trimmomatic (v039), SAMtools (v1.2), Integrative Genomics Viewer (v2.11.9), AlphaFold2 database (https://alphafold.ebi.ac.uk), seeSAR v12.1 (BioSoveIT), Pymol v2.5.2 (Schrödinger), JalView (v2.11.2.6).

For manuscripts utilizing custom algorithms or software that are central to the research but not yet described in published literature, software must be made available to editors and reviewers. We strongly encourage code deposition in a community repository (e.g. GitHub). See the Nature Portfolio guidelines for submitting code & software for further information.

Data

Policy information about availability of data

All manuscripts must include a data availability statement. This statement should provide the following information, where applicable:

- Accession codes, unique identifiers, or web links for publicly available datasets
- A description of any restrictions on data availability
- For clinical datasets or third party data, please ensure that the statement adheres to our policy

All data is available in the Article, the Supplementary Information, and also from the corresponding author on reasonable request. Gel source images are shown in Supplementary Fig. 2. All source data is provided in this paper. Human cancer cell line data was mined from DepMap (https://depmap.org/portal/) or COSMIC

(https://cancer.sanger.ac.uk/cosmic) databases. Murine cancer cell line data was mined from TISIMO database (http://tismo.cistrome.org). The sequence data from this study has been submitted to NCBI BioProject (https://www.ncbi.nlm.nih.gov/bioproject) under BioProject ID PRJNA942499.

Human research participants

Policy information about studies involving human research participants and Sex and Gender in Research.

Reporting on sex and gender	n.a.
Population characteristics	n.a.
Recruitment	n.a.
Ethics oversight	n.a.

Note that full information on the approval of the study protocol must also be provided in the manuscript.

Field-specific reporting

Please select the one below that is the best fit for your research. If you are not sure, read the appropriate sections before making your selection.

☒ Life sciences ☐ Behavioural & social sciences ☐ Ecological, evolutionary & environmental sciences

For a reference copy of the document with all sections, see nature.com/documents/nr-reporting-summary-flat.pdf

Life sciences study design

All studies must disclose on these points even when the disclosure is negative.

Sample size	Sample sizes in vitro experiments were determined based on the numbers required to achieve statistical significance using indicated statistics, as well as considering of previous publications on similar experiments (PMID: 31634899 and 35922516).
Data exclusions	No data exclusions.
Replication	The experimental findings were reproduced as validated by at least two independent experiment in Fig. 1-5 and Extended Data Fig. 2-7 except for the mutational analysis.
Randomization	Randomization is not relevant to the in vitro experiments since cells come in millions of populations and are automatically randomized and seeded to different wells for treatment.
Blinding	In the in vitro experiments, the investigators were not blinded, which is standard in this type of study due to the multiple steps involved that require precise operations for accuracy and precision precluding blinding to experimental variables.

Reporting for specific materials, systems and methods

We require information from authors about some types of materials, experimental systems and methods used in many studies. Here, indicate whether each material, system or method listed is relevant to your study. If you are not sure if a list item applies to your research, read the appropriate section before selecting a response.

Materials & experimental systems

n/a	Involved in the study	
☐	☒	Antibodies
☐	☒	Eukaryotic cell lines
☒	☐	Palaeontology and archaeology
☒	☐	Animals and other organisms
☒	☐	Clinical data
☒	☐	Dual use research of concern

Methods

n/a	Involved in the study	
☒	☐	ChIP-seq
☐	☒	Flow cytometry
☒	☐	MRI-based neuroimaging

Antibodies

Antibodies used	GPX4 (1:1000 for WB, ab125066, Abcam), human FSP1 (1:1000, sc-377120, Santa Cruz Biotechnology), human FSP1(1:5 for WB, clone AIFM2 6D8, rat IgG2a, developed in-house: available from Sigma,

Cat#MABC1638-25UL), mouse FSP1 (1:100 for WB, clone AIFM2 1A1 rat IgG2a, developed in-house), mouse FSP1(1:5 for WB, clone AIFM2 14D7 IgG2b, developed in-house), β-actin-HRP (1:50000, A3854, Sigma-Aldrich), valosin containing protein (VCP, 1:10000, ab11433 or ab109240, Abcam), HA tag (YPYDVPDYA, 1:1000 for WB, clone 3F10 rat IgG1, developed in-house) were used in this study. The appropriate secondary antibodies (1:1000-5000, Cell Signaling, Cat#7074S for rabbit; 7076S for mouse, and 1:1000 for anti-rat IgG1b and 2a/b, developed in-house) diluted in 5% skim milk in TBS-T.

Validation

GPX4 (ab125066), VCP (ab11433 or ab109240), human FSP1 (sc-377120), HA tag (clone 3F10), β-actin-HRP (A3854) antibodies were validated for WB using mouse and human cell samples in previous publications (PMID: 35922516, 31634899, and 27842070).
FSP1 antibody (clone AIFM2 1A1/6D8 rat IgG2a, and clone AIFM2 14D7 IgG2b, developed in-house) has been validated for WB in previous study (PMID: 35922516).

Eukaryotic cell lines

Policy information about cell lines and Sex and Gender in Research

Cell line source(s)

4-OH-TAM-inducible Gpx4-/- murine immortalized fibroblasts (Pfa1) were established in our lab as reported previously (PMID: 18762024). HT-1080 (CCL-121), HEK293T (CRL-3216), 786-O (CRL-1932), A375 (CRL-1619), B16F10 (CRL-6475), LLC (CRL-1642), MDA-MB-436 (HTB-130), SW620 (CCL-227), NCI-H460 (HTB-177) and 4T1 (CRL-2539) cells were obtained from ATCC. MC38 cells (SCC172) and SKOV3 (91091004) were obtained from Sigma-Aldrich. HEC151 cells (JCRB1122-A) were obtained from Tebubio. Rat1 cells (available from Thermo Fisher) were kindly gifted from Medizinische Hochschule Hannover. Huh7 cells (available from Thermo Fisher) were kindly gifted from Dr. Robert Schneider, Helmholtz Munich. MC38 cells (available from Sigma) were kindly gifted from Dr. Patrizia Agostinis (KU Leuven, Belgium).

Authentication

None of the cell lines used were authenticated.

Mycoplasma contamination

All cell lines were tested negative for mycoplasma contamination.

Commonly misidentified lines
(See ICLAC register)

No commonly misidentified cell lines were used.

Flow Cytometry

Plots

Confirm that:

☒ The axis labels state the marker and fluorochrome used (e.g. CD4-FITC).

☒ The axis scales are clearly visible. Include numbers along axes only for bottom left plot of group (a 'group' is an analysis of identical markers).

☒ All plots are contour plots with outliers or pseudocolor plots.

☒ A numerical value for number of cells or percentage (with statistics) is provided.

Methodology

Sample preparation

100,000 cells per well were seeded on a 12-well plate one day prior to the experiments. On the next day, cells were treated with 2.5 µM icFSP1 for 3 h, and then incubated with 1.5 µM C11-BODIPY 581/591 (Invitrogen, Cat#D3861) for 30 min in a 5% CO2 atmosphere at 37°C. Subsequently, cells were washed by PBS once and trypsinized, and then resuspended in 500 µL PBS. Cells were passed through a 40 µm cell strainer and analyzed by a flow cytometer (CytoFLEX, Beckman Coulter) with a 488-nm laser for excitation. Data was collected from the FITC detector (for the oxidized form of BODIPY) with a 525/40nm bandpass filter and from the PE detector (for the reduced form of BODIPY) with a 585/42 nm bandpass filter.

Instrument

CytoFLEX (Beckman Coulter)

Software

CytExpert v2.4 was used for data collection. FlowJo v10 was used for data analysis.

Cell population abundance

At least 10,000 cells were analyzed for each sample.

Gating strategy

Cell populations were separated from cellular debris using FSC and SSC.

☒ Tick this box to confirm that a figure exemplifying the gating strategy is provided in the Supplementary Information.
